# Low Gain Servo Control During the Kohnstamm Phenomenon Reveals Dissociation Between Low-Level Control Mechanisms for Involuntary vs. Voluntary Arm Movements

**DOI:** 10.3389/fnbeh.2018.00113

**Published:** 2018-05-30

**Authors:** Jack De Havas, Sho Ito, Patrick Haggard, Hiroaki Gomi

**Affiliations:** ^1^NTT Communication Science Laboratories, Nippon Telegraph and Telephone Corporation, Atsugi, Japan; ^2^Institute of Cognitive Neuroscience, University College London, London, United Kingdom; ^3^International Research Fellow of Japan Society for the Promotion of Science, Tokyo, Japan

**Keywords:** kohnstamm phenomenon, involuntary movement, aftercontraction, servo-control, voluntary movement, muscle afferents, electromyography

## Abstract

The Kohnstamm phenomenon is a prolonged involuntary aftercontraction following a sustained voluntary isometric muscle contraction. The control principles of the Kohnstamm have been investigated using mechanical perturbations, but previous studies could not dissociate sensorimotor responses to perturbation from effects of gravity. We induced a horizontal, gravity-independent Kohnstamm movement around the shoulder joint, and applied resistive or assistive torques of 0.5 Nm after 20° angular displacement. A No perturbation control condition was included. Further, participants made velocity-matched *voluntary* movements, with or without similar perturbations, yielding a 2 × 3 factorial design. Resistive perturbations produced an increase in agonist electromyography (EMG), in both Kohnstamm and voluntary movements, while assistive perturbations produced a decrease. While overall Kohnstamm EMGs were greater than voluntary EMGs, the EMG responses to perturbation, when expressed as a percentage of unperturbed EMG activity, were significantly *smaller* during Kohnstamm movements than during voluntary movements. The results suggest that the Kohnstamm aftercontraction involves a central drive, coupled with low-gain servo control by a negative feedback loop between afferent input and a central motor command. The combination of strong efferent drive with low reflex gain may characterize involuntary control of postural muscles. Our results question traditional accounts involving purely reflexive mechanisms of postural maintenance. They also question existing high-gain, peripheral accounts of the Kohnstamm phenomenon, as well as accounts involving a central adaptation interacting with muscle receptors via a positive force feedback loop.

## Introduction

Postural control involves maintaining the stability of the body by appropriately modulating the efferent and afferent signals that drive skeleto-muscular contractions in the face of external perturbations (Davidoff, 1992). For example, high muscle reactivity to environmental events may indicate high gain on the afferent arm of a sensorimotor control loop. Such responses are modulated by factors such as task, context, velocity of movement and level of motor drive (Rothwell et al., 1980; Traub et al., 1980; Scott, 2012).

Appropriate setting of reflex gains also has a key role in simple models of voluntary movement. On one view, a desired muscle length is set by a descending command. The gain of the stretch reflex then triggers negative position feedback to adjust the ongoing movement (Marsden et al., 1975, 1976, 1977). Both classical and recent models of voluntary action assume a crucial role of “follow-up” reflex servo mechanisms (Marsden et al., 1976; Friston et al., 2010).

However, it remains unclear whether involuntary movements also show comparable modulation of afferent gain. Most experimental models of involuntary movement involve brief reflex responses (Matthews, 1991)—superimposing an additional perturbation onto such brief involuntary contractions, in order to measure reflex gains, would be difficult. Therefore, a fair experimental test comparing responses to perturbation during voluntary movements and physically-matched involuntary movements is of key importance for clarifying the distinctive features and control principles that differ between voluntary and involuntary movements.

The Kohnstamm phenomenon, however, provides a useful experimental model to investigate differences between voluntary and involuntary movements (Duclos et al., 2007; De Havas et al., 2017). Classically, participants hold the arm straight and vertical by their side and push outwards against a solid surface for 30 s, by a strong voluntary contraction of the lateral deltoid muscle. Upon relaxation the arm rises involuntarily (Kohnstamm, 1915; De Havas et al., 2016). This aftercontraction is electromyographically (EMG) similar to a slow voluntary movement (Mathis et al., 1996) and can be elicited in many skeletal muscles (Forbes et al., 1926). Central (Salmon, 1925; Sapirstein et al., 1938) and peripheral (Hagbarth and Nordin, 1998) accounts of the adaptation underlying the Kohnstamm phenomenon have been proposed. Peripheral accounts emphasize an increase in afferent reflex gain due to muscle thixotropy. Kohnstamm induction promotes the formation of stable actin-myosin cross bridges in intrafusal muscle fibers. The resulting shortness and stiffness of the intrafusal muscle fibers causes muscle spindles to be hypersensitive when stretched, resulting in a sustained extrafusal muscle contraction via spinal reflex pathways (Gregory et al., 1988; Hagbarth and Nordin, 1998). As such, the theory predicts a large EMG response to the perturbation of Kohnstamm aftercontractions. However, few studies have explored such perturbations.

One previous study used a counterweight to vary the load during the involuntary arm movement (Parkinson and McDonagh, 2006). Aftercontraction EMG reduced as the counterweight decreased muscle load. A positive force feedback model was proposed, with putative firing rates of Golgi tendon organs causing an excitatory drive to motor neurons. However, the results could also be explained by negative position or velocity feedback from muscle spindles, since decreased loading would reduce muscle stretch and hence spindle-evoked drive to motor neurons. These two models predict subtly different responses to perturbations, since negative feedback loops have a corrective effect, while positive feedback loops produce explosive action (Latash, 2008a). However, previous studies (Parkinson and McDonagh, 2006; De Havas et al., 2015) involved vertical involuntary movements, meaning muscle load varied continuously due to gravity. Thus, the active neural mechanisms that resist perturbation could not be easily isolated.

We therefore investigated the response of the Kohnstamm phenomenon to perturbations independent of gravity, apparently for the first time. We used a horizontal movement of a single joint, and compared EMG responses to ramped, sustained force perturbations during involuntary Kohnstamm movements, and velocity-matched voluntary movements. This allowed us to compare the sensorimotor gain of the two types of movements directly.

## Materials and Methods

### Participants

A total of 39 participants were recruited (13 female; age: mean = 31.62, SD = 5.34). Since the basic Kohnstamm phenomenon is absent in around 30% of healthy participants (Adamson and McDonagh, 2004; Ghosh et al., 2014), we first screened participants for presence of a Kohnstamm phenomenon in the lateral deltoid muscle. Fourteen participants showed no discernible Kohnstamm phenomenon in a screening test while standing (arm elevation was absent). They were therefore excluded, leaving 25 participants (Female = 8; age: mean = 32.32, SD = 5.47). Previous authors have advocated such screening of participants and mentioned that the failure of some participants to demonstrate the Kohnstamm phenomenon could be due to anxiety induced by the testing procedure (Craske and Craske, 1985). The horizontal movements studied here involve the posterior deltoid, which has not been investigated in any previous Kohnstamm study to our knowledge. Previous studies using other muscle groups have reported failures to achieve the Kohnstamm phenomenon in up to 40% of participants tested (Ghafouri et al., 1998). We then tested the posterior deltoid and found that four further participants did not display any discernible Kohnstamm phenomenon in the posterior deltoid. They were excluded, leaving 21 participants (Female = 7; age: mean = 32.48, SD = 5.14). Of these, we found that our resistive perturbations completely arrested Kohnstamm arm movements in six participants, indicating that for these participants the perturbation was strong enough to act like a rigid obstacle, as has been used in previous experiments (De Havas et al., 2015). To clearly characterize the on-going movement and EMG effects following perturbations these participants were excluded from the main analysis. Therefore, perturbation responses between voluntary and Kohnstamm movements of 15 participants (four Female; age: mean = 32.27, SD = 5.56) were compared in this study.

Experiments were undertaken with the understanding and written consent of each participant in accordance with the Code of Ethics of the World Medical Association (Declaration of Helsinki), and with approval of the local NTT BRL ethical committee. No adverse events occurred during the experiment.

### Equipment

**Figure 1:**
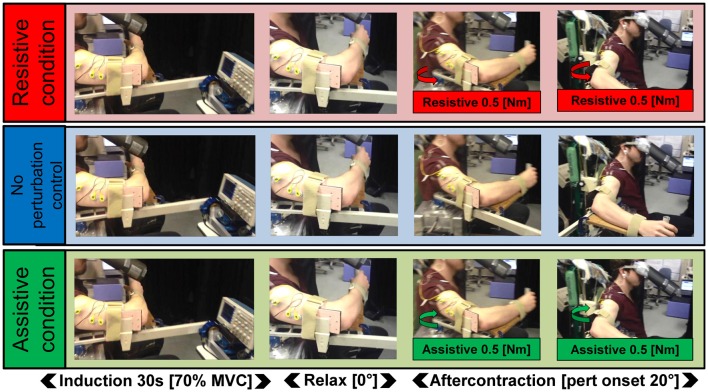
Schematic of the task. In all three Kohnstamm perturbation conditions, participants maintained a constant isometric contraction of the right posterior deltoid for 30 s (70% MVC). They then relaxed and the aftercontraction began. In the Resistive perturbation condition, a torque was applied by the manipulandum motor in the opposite direction to the movement (0.5 Nm; 250 ms linear ramp) once the angular displacement of the arm reached 20°. In the Assistive perturbation condition, a torque was applied by the manipulandum motor in the same direction as the movement (0.5 Nm; 250 ms linear ramp) once the angular displacement of the arm reached 20°. In the No perturbation control condition, no torque was applied.

Electromyography (EMG) was recorded from bipolar, surface electrodes (Ag-AgCl disposable electrode, GE Healthcare Japan, Tokyo, Japan) placed over the middle of the right posterior deltoid, parallel to the orientation of the muscle fibers. In a subset of participants, additional electrodes were also placed on the right pectoralis (*n* = 11) and the right triceps long head (*n* = 9). The electrodes were connected to an amplifier (MME-3116, Nihon Kohden, Tokyo, Japan), which was controlled via custom scripts. EMG data were sampled and recorded at 4000 Hz. A fully adjustable chair positioned each participant relative to a single-joint manipulandum (Figure 1; Max torque 6.8 Nm servo-bandwidth 2000 Hz). The position of the manipulandum was calculated via a rotary encoder (resolution of 0.0055°) and output (D/A converted) at 2000 Hz. Torque was measured with a 6-axis force sensor (UFS-3012A25, Nitta, Osaka, Japan). The manipulandum was controlled via custom MATLAB (2007b) scripts. The manipulandum had a strip of wood (60 × 10 × 2 cm) clamped at a right angle with an upwards pointing handle at one end. This was to support the participant’s forearm and was fully adjustable. A custom-built, rigid pushing surface was clamped to the manipulandum. This was adjustable so that the participant could comfortably push against it. On the opposite side of the manipulandum to the participant was a force sensor (UFS-3012A15, Nitta, Osaka, Japan) mounted to a moveable, rigid beam and located at a distance of 0.5 m from the rotation center of the manipulandum. This was positioned such that the force sensor would register the amount of torque being generated by the participant during isometric Kohnstamm inductions. This information was relayed to participants via an oscilloscope (TDS2004C, Tektronix Inc., Oregon, USA) positioned at eye level in front of them. They could thus regulate the Kohnstamm-inducing voluntary contraction. Participants wore goggles with a cardboard cone (length = 45 cm) to prevent them from seeing their arm. Unwanted movement of the right arm was prevented via two adjustable straps on the forearm and upper arm, fixing the participant with respect to the apparatus. A flat screen monitor (19-inch LCD, 800 × 600 pixels, 60 Hz refresh rate) was positioned in front of the participants to provide visual feedback of arm position for learning to make voluntary movements matched to Kohnstamm movements. Visual feedback was controlled by Cogent Graphics (John Romaya, Wellcome Trust Centre for Neuroimaging and Institute of Cognitive Neuroscience development team, UCL) in MATLAB (2007b). Analog signals (EMG, position, toque during induction and aftercontraction) were sampled and stored at 4000 Hz via custom-made software (MATLAB, 2007b). The experimental set up is shown in Figure 1.

### Procedure

Participants were seated throughout the experiment wearing goggles that prevented any view of their right arm. The chair was adjusted for each participant such that the right arm rested on the manipulandum, with the elbow bent at 90°, the shoulder above the center of rotation and the arm horizontal to the ground. Shoulder angle at the start of each trial was 0° relative to the midline of the trunk. Unwanted movement was prevented by strapping the forearm and upper arm to the manipulandum. This ensured that elbow angle remained constant throughout the experiment. The handle rested between thumb and forefinger. Participants were instructed not to grip the handle, as this might also influence contraction of proximal muscles. Tilt and rotation of the chair were adjusted until the participant’s arm remained in the start position when relaxed. This prevented any movement occurring as a result of the tension on the shoulder or the release of antagonist contraction.

Participants completed a 5 s, 100% MVC isometric contraction of the posterior deltoid muscle (agonist) in the home position, by pushing outwards against the rigid elbow support. The oscilloscope was then set to display 70% of this value as the target force level for the voluntary contractions used to induce the Kohnstamm. If EMG data was being recorded from the pectoralis (antagonist) and triceps long head muscle, 5 s, 100% MVC isometric contractions were also recorded for these muscles.

A tone signaled the start of each Kohnstamm trial. Participants maintained a 70% MVC isometric contraction of the lateral deltoid by pushing outwards against the support. Target force and actual force were displayed continuously on the oscilloscope. After 30 s a tone signaled that they should stop pushing and relax. As soon as the force level reached zero, the experimenter rotated the support and attached force sensor away, allowing the arm to move freely. This ensured that the shoulder angle remained at 0° immediately after the induction period, prior to the onset of any Kohnstamm aftercontraction. This could be done prior to onset of involuntary movement, owing to the latent period of muscle silence that occurs for 1–3 s in the Kohnstamm phenomenon (Csiky, 1915; Pinkhof, 1922; Kozhina et al., 1996; Parkinson and McDonagh, 2006). An aftercontraction of the posterior deltoid then occurred causing an involuntary movement of the arm. The shoulder was free to rotate 100°. Participants were instructed to remain relaxed and not attempt to move the arm voluntarily.

In the *No perturbation* control condition, the arm was allowed to move freely. However, in the *Resistive* perturbation condition a constant torque of 0.5 Nm was applied at the shoulder in the opposite direction to movement, once the arm reached 20° of angular displacement. In the *Assistive* perturbation condition, the same torque was applied in the direction of movement (Figure 1). A ramp was used in both cases, such that the applied torque increased linearly over a duration of 250 ms. This ensured arm movement was smooth. Importantly, the perturbation was not felt as rigid obstacle, as this could induce the kind of “afferent resetting” seen in previous studies (De Havas et al., 2015). We planned six trials, two for each of the three conditions (No perturbation control condition, Assistive perturbation condition, Resistive perturbation condition). Trial order was ABCCBA, counterbalanced across participants. Participants were always naïve to perturbation condition. The Kohnstamm phenomenon is known to be highly variable (Brice and McDonagh, 2001; Hagbarth and Nordin, 1998; Salmon, 1916, 1925). We therefore repeated trials where no clear Kohnstamm movement was detected visually by the experimenter. Because of these occasional repetitions, the actual number of trials undertaken by each participant was therefore slightly higher than the intended number of 6 (Mean = 6.67, SD = 0.98). If trials had to be repeated, we maintained the randomization process by re-adjusting trial order, so that the mean position of trials within the order of the experiment did not differ across perturbation conditions. Average position of trials did not significantly differ across perturbation conditions: No perturbation control (Mean = 3.77, SD = 0.42) vs. Resistive (Mean = 4.23, SD = 1.45) vs. Assistive perturbations (Mean = 3.53, SD = 1.03; *F*_(2,28)_ = 2.479, *p* = 0.102). After every trial there was a rest period of 7 min to minimize fatigue and long-lasting motor post-effects (Hutton et al., 1987; Duclos et al., 2004).

Each participant’s Kohnstamm No perturbation control trials were used as models for their velocity-matched voluntary movements. This was done separately for each of the two trials. First, one of the two Kohnstamm No perturbation control trials was randomly selected to create a template for half of each participant’s voluntary replication trials. Participants heard a tone signaling that the movement was to begin in 3 s. They then saw the trajectory of the Kohnstamm control trial represented on the screen as a moving dot. They replicated the previous involuntary movement in real-time with a voluntary contraction of the posterior deltoid. Position of the arm was displayed continuously as a line of hollow circles. Participants completed 10 voluntary practice trials, followed by 12 voluntary trials in which no visual feedback was given. We included this extensive voluntary practice to ensure that the voluntary movements during experimental trials were fluent, and participants had learned to reproduce Kohnstamm-like trajectories without co-contraction. As with the Kohnstamm trials, these voluntary no-feedback trials could be perturbed at random, with Resistive or Assistive perturbations, or No perturbation control condition. Perturbations were applied in exactly the same manner for voluntary replications as for Kohnstamm movements. Participants were not told about the perturbations and simply instructed to complete each movement. Interposed with these voluntary trials were occasional trials in which visual feedback was given to ensure replication accuracy was maintained. Trials with visual feedback never included perturbations and were not analyzed. This entire replication process was then repeated using the second Kohnstamm control trial as a visual template, resulting in a total of 24 voluntary movement trials. The experiment lasted ~2.5 h.

### Analysis

**Figure 2:**
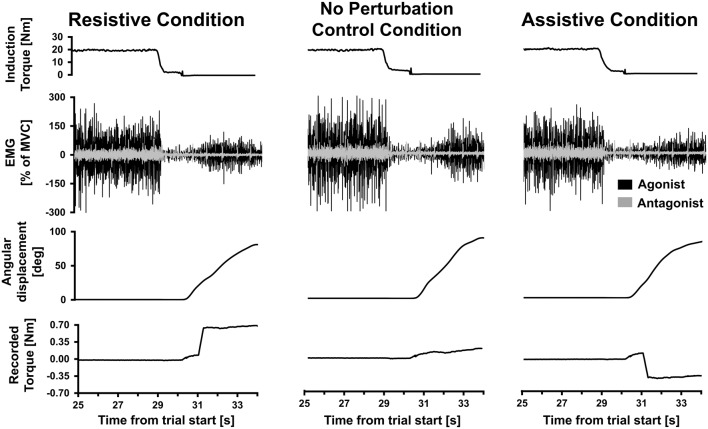
Single trial data. Data from three Kohnstamm movement trials from a single representative participant, belonging to the Resistive perturbation, No perturbation control and Assistive perturbation conditions. Last 5 s of the 30 s isometric induction contraction is shown, followed by a brief latent period of ~1 s and then the aftercontraction. Note that induction torque was equivalent across trials. During aftercontraction agonist (posterior deltoid) electromyography (EMG) increased in amplitude as angular displacement of the shoulder increased. Antagonist (pectoralis) EMG was flat throughout the aftercontraction (regular spikes shown were from heart beat artifact). In the Resistive perturbation condition trial, a torque was applied in the opposite direction to the movement once angular displacement reached 20° (0.5 Nm; 250 ms linear ramp). In the Assistive perturbation condition trial, a torque was applied in the same direction as the movement once angular displacement reached 20° (0.5 Nm; 250 ms linear ramp). The lower traces show the torque recorded at the shoulder manipulandum: note the abrupt changes in torque due to the perturbations.

Kohnstamm trials were only included in the analysis if the arm continued to move for at least 500 ms after the perturbation onset. Examples of trials from each perturbation condition can be seen in Figure 2. If arm movement stopped within this window the trial was repeated (see “Procedure” section). If the perturbation again stopped the arm on the repeat trial, we did not continue indefinitely because the total number of trials is limited by fatigue (Danielopolu et al., 1921; Allen and O’Donoghue, 1927; Allen, 1937; Zigler et al., 1948). Of the 15 participants included in the main analysis, two participants only achieved a single trial in the Resistive perturbation condition for this reason. Additionally, technical errors led to loss of one Resistive trial, and one Assistive trial, each in a different participant. Angular velocity was computed by calculating one-sample differences and then low-pass fourth-order Butterworth filtered at 80 Hz. For the voluntary replication movements, six trials (two per condition) were selected for each participant. We selected the three trials (1 per condition) that had pre-perturbation velocity closest to each of the two Kohnstamm No perturbation control trials used as templates. This was done by calculating mean SSE from the voluntary trial velocity compared to the Kohnstamm control trial velocity, between 10 and 20° of angular displacement.

The force sensor signal was low-pass fourth-order zero-phase Butterworth filtered at 30 Hz. EMG was band-pass filtered (zero-phase digital filtering; 10–500 Hz) and rectified before being smoothed (4 Hz). A 1 s window was selected for the purposes of displaying the data, centered on the onset of the perturbation. For the agonist muscle, two alternative forms of normalization were used. The first involved normalizing to each participant’s MVC (EMG as mean % MVC across the three perturbation conditions). This standard form of normalization was also used for the antagonist muscle (pectoralis) and triceps long head muscle. Since background level of agonist EMG is known to influence the size of reflex responses (Matthews, 1986; Toft et al., 1989), an alternative normalization was also used. Each participant’s Resistive and Assistive perturbation condition agonist EMG was normalized to their No perturbation control condition agonist EMG (% EMG change relative to No perturbation control condition, across the two perturbation conditions). So, for example, in the case of the resistive perturbation condition the following formula was applied to every time point, separately for each participant: [Resistive perturbation condition EMG]/[No perturbation control condition EMG] × 100%. This normalization was performed separately for Kohnstamm and voluntary movements.

Mean agonist EMG, antagonist EMG, triceps long head EMG, torque, angular displacement and velocity were calculated during an analysis window of 200–400 ms post-perturbation. 2 (Movement type: Kohnstamm vs. Voluntary) × 3 (Condition: No perturbation control vs. Resistive perturbation vs. Assistive perturbation) within subjects ANOVA were conducted.

Visual inspection of the velocity data showed a change in the direction of the group level response to perturbation, occurring around 400 ms post-perturbation. To explore this, an additional 400–500 ms time window was selected for the analysis of movement velocity. Because the characteristic feature of reflex responses is a near-linear increase or decrease in velocity during the relevant time period (Crevecoeur et al., 2012; Bourke et al., 2015), linear regression lines were applied to calculate the slope for each participant’s individual mean velocity data in this time window, separately for each perturbation condition and for Voluntary vs. Kohnstamm movements. Mean slope values were compared using 2 × 3 within subjects ANOVA as before.

For the “EMG % of no perturbation control” normalization, a 2 (Movement type: Kohnstamm vs. Voluntary) × 2 (Perturbation condition: Resistive vs. Assistive) within subjects ANOVA was conducted on the agonist EMG data, based on mean values during the same time window (200–400 ms post-perturbation). In addition, a linear trend analysis of the EMG data (% of no perturbation control) was conducted on the entire time window (0–500 ms post-perturbation) for both Kohnstamm and Voluntary movement types, to determine the overall trend in the data for each subject. Two-by-two within subjects ANOVAs were conducted as above.

The Kohnstamm induction period was analyzed to determine if muscle fatigue was present during Kohnstamm phenomenon. We selected the first and last Kohnstamm trial of each participant and excluded the first and last 3 s of the induction period, leaving a 24 s window. We then graphed the induction period EEG (resampled to 400 Hz, filtered, rectified and 4 Hz smoothed; % of MVC) and force (30 Hz low-pass filtered; % of MVC) during this time window. The relationship between force and EMG was also calculated. One participant was excluded from the analysis because EMG was saturated during the induction due to the amplifier gain being set too high. Statistical analysis was performed on this mean EMG/Force ratio during the first and last 1 s of the 24 s analysis window. A two-by-two within subjects ANOVA was conducted with the factors of Time (first vs last 1 s of induction) and Kohnstamm trial (first trial vs. last trial). If muscle fatigue were present, the ratio of EMG to force would increase both during each sustained isometric contraction, and from the first to the last of these contractions (Bigland-Ritchie, 1981).

## Results

### Perturbation Responses During Involuntary and Voluntary Movements

**Figure 3:**
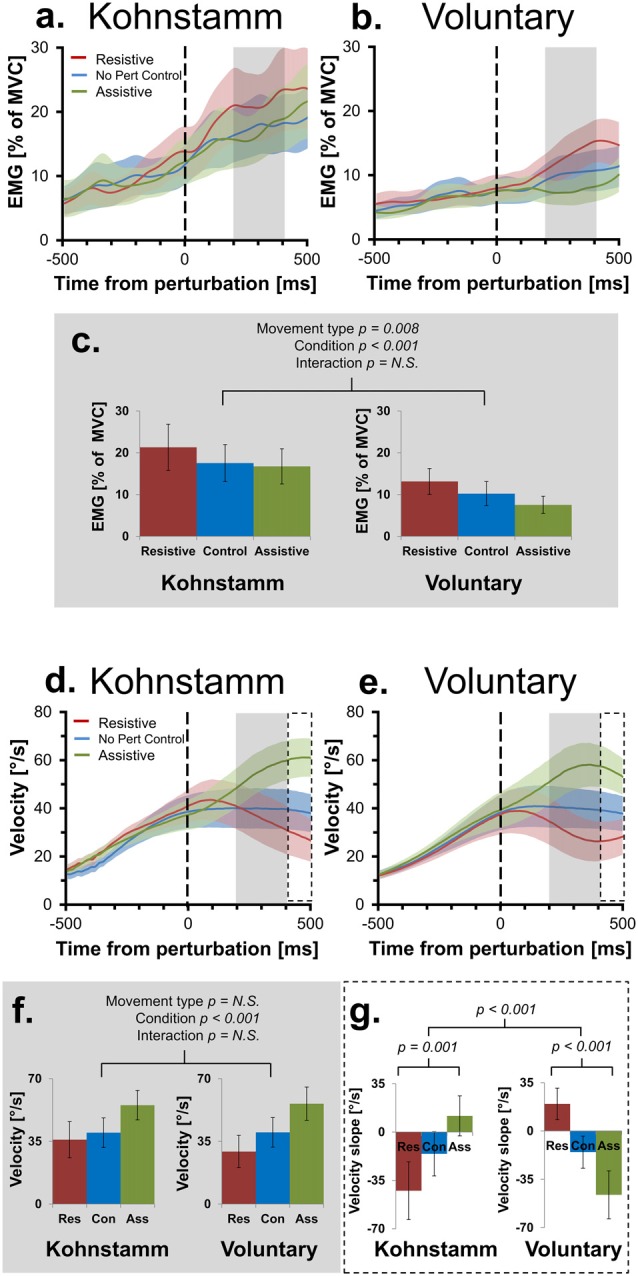
Mean smoothed agonist EMG and velocity of movement across movement types and perturbation conditions. Agonist smoothed group EMG (% MVC) across conditions, from 500 ms prior to onset of perturbation to 500 ms post-perturbation, for Kohnstamm **(A)** and Voluntary movements **(B)**. Mean EMG 200–400 ms post-perturbation **(C)**. There was significantly higher EMG during Kohnstamm movements than Voluntary movements during this time window. EMG increased in the Resistive perturbation condition and decreased in the Assistive perturbation condition, relative to the No perturbation control condition. This change in EMG was significant across the two types of movement. Velocity of angular displacement during the same time window for Kohnstamm **(D)** and Voluntary movements **(E)**. Mean velocity 200–400 ms post-perturbation **(F)**. Velocity decreased in the Resistive perturbation condition and increased in the Assistive perturbation condition, relative to the No perturbation control condition (200–400 ms post-perturbation). This change in velocity was significant across the two types of movement. Mean slope of velocity across participants (400–500 ms post-perturbation) showed the opposite pattern of results when comparing Kohnstamm to Voluntary movements **(G)**.

As expected, the resistive perturbation produced an increase in agonist EMG during both voluntary and Kohnstamm movements, while the assistive perturbation produced a decrease, compared to the No perturbation control condition (Figures 3A–C). This produced a significant main effect of Perturbation Condition (*F*_(2,28)_ = 10.349, *p* < 0.001). There was no Movement type × Perturbation Condition interaction (*F*_(2,28)_ = 0.676, *p* = 0.517), indicating that the responses to perturbation were similar for Kohnstamm and voluntary movements. There was a significant main effect of Movement type (*F*_(1,14)_ = 9.377, *p* = 0.008) on agonist EMG, with EMG being higher during Kohnstamm movements than voluntary movements.

### Perturbation Response *Smaller* During Kohnstamm Than Voluntary Movements After Controlling for EMG in the No Perturbation Control Condition

**Figure 4:**
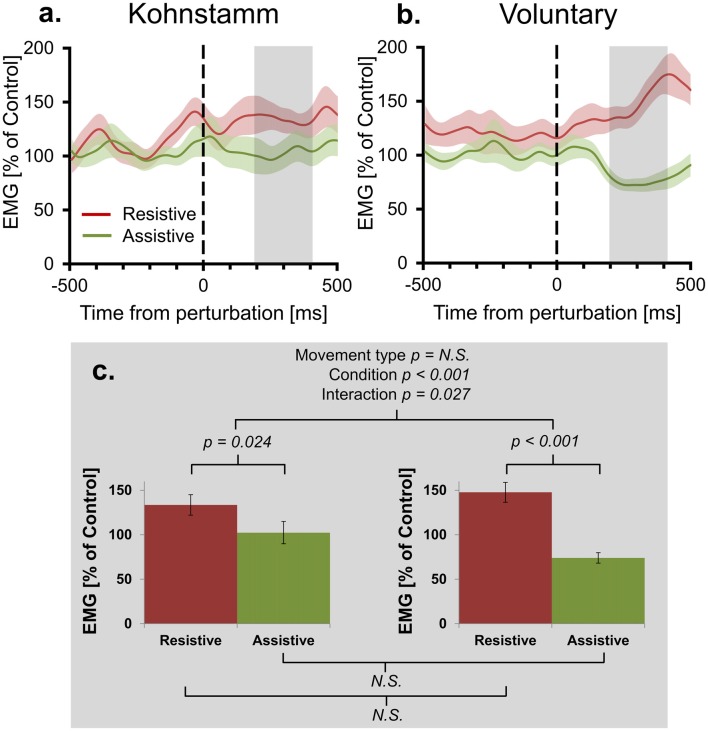
Mean agonist EMG in Resistive and Assistive conditions normalized to No perturbation control condition. Agonist smoothed group EMG (% No perturbation control condition) across conditions, from 500 ms prior to onset of perturbation to 500 ms post-perturbation, for Kohnstamm **(A)** and Voluntary movements **(B)**. Note larger difference between perturbation conditions for Voluntary movements. Mean EMG (% No perturbation control condition) 200–400 ms post-perturbation **(C)**. There was no difference in overall EMG level across movement types. There was larger EMG in the Resistive perturbation condition than in the Assistive perturbation condition across movement types. This difference was significantly larger for Voluntary movements than Kohnstamm movements.

Figure 3 shows that background EMG levels during Kohnstamm were high but also variable. Responses to perturbations are classically proportional to the background level of EMG (Matthews, 1986; Toft et al., 1989). Indeed, we found that for voluntary movements there was a significant positive correlation (*N* = 15, *r* = 0.76, *p* < 0.01) across participants between the size of the EMG (% MVC) perturbation response (Resistive perturbation condition minus Assistive perturbation condition, 200–400 ms post perturbation) and the No perturbation control condition EMG in the same time window. We therefore compared responses to perturbation of Kohnstamm and Voluntary movements after controlling for the respective background level of EMG. Agonist EMG was first expressed as a percentage of each participants’ EMG in the same time window of the No perturbation control condition (instead of the conventional normalization to MVC; Figure 4). A 2 × 2 ANOVA showed that mean EMG did not significantly differ cross Movement types (*F*_(1,14)_ = 0.242, *p* = 0.630). The perturbation still decreased EMG in the Assistive perturbation condition and increased it in the Resistive perturbation condition, as evidenced by a significant main effect of Condition (*F*_(1,14)_ = 31.535, *p* < 0.001). Importantly, the effect of perturbation type was larger for Voluntary than for Kohnstamm movements, as evidenced by a significant Movement type by Perturbation condition interaction (*F*_(1,14)_ = 6.146, *p* = 0.027). Planned comparisons showed that Resistive perturbation condition EMG was higher than Assistive perturbation condition EMG for Kohnstamm movements (*t*_(14)_ = 2.54, *p* = 0.024) and for Voluntary movements (*t*_(14)_ = 5.641, *p* < 0.001), showing a perturbation response in both cases. However, the interaction arose because Kohnstamm responses were weaker than Voluntary responses after the normalization procedure.

### Linear Trend Analysis Does Not Support Positive Force Feedback Model of Kohnstamm Phenomenon

Positive force feedback models of the Kohnstamm phenomenon predict that there will be a continuous increase in EMG for Kohnstamm movements in the Resistive perturbation condition and a continuous decrease in the Assistive perturbation condition, for as long as the perturbation remains present. To test this, we fitted linear trends to participants’ mean EMG (% of No perturbation control condition) across the entire analysis window (0–500 ms post-perturbation) for Resistive and Assistive perturbation conditions during both Kohnstamm and Voluntary movements. For Voluntary movements the mean slope of trend lines was positive in the Resistive perturbation condition (Mean = 118.629, SD = 128.697) and negative in the Assistive perturbation condition (Mean = −65.183, SD = 78.973). For Kohnstamm movements the mean slope of trend lines was slightly positive in the Resistive perturbation condition (Mean = 25.67, SD = 128.125) and close to zero in the Assistive perturbation condition (Mean = −0.768, SD = 145.163). This resulted in no significant main effect of Movement type (*F*_(1,14)_ = 0.114, *p* = 0.741), but a significant main effect of Perturbation condition (*F*_(1,14)_ = 14.287, *p* = 0.002) and a significant Movement type by Perturbation condition interaction (*F*_(1,14)_ = 9.375, *p* = 0.008). Planned comparisons showed that the significant interaction was due to a significant difference between the Resistive and Assistive perturbation condition for Voluntary movements (*t*_(14)_ = 6.374, *p* < 0.001) and a no significant difference for Kohnstamm movements (*t*_(14)_ = 0.586, *p* = 0.567). There was no significant difference for the Resistive perturbation condition between Kohnstamm and Voluntary movements (*t*_(14)_ = −1.68, *p* = 0.115), nor for the Assistive perturbation condition (*t*_(14)_ = 1.503, *p* = 0.155).

We further evaluated the presence of an overall trend in the data by testing mean EMG (% of No perturbation control condition) slope for each perturbation condition, in each movement type, against zero. A Bonferroni corrected significance threshold of 0.0125 was used. For Voluntary movements, mean slope of EMG (0–500 ms post-perturbation) was significantly higher than 0 in the Resistive perturbation condition (*t*_(14)_ = 3.57, *p* = 0.003) and significantly lower than 0 in the Assistive perturbation condition (*t*_(14)_ = −3.197, *p* = 0.006). However, for Kohnstamm no difference to 0 was found for either Resistive (*t*_(14)_ = 0.776, *p* = 0.451) or Assistive perturbation conditions (*t*_(14)_ = −0.02, *p* = 0.984). Taken together, the results show that there was no sustained upwards trend in Kohnstamm aftercontraction EMG in response to an increased load on the muscle, and no sustained downward trend in response to a decreased load. Instead, the EMG response to perturbations during the Kohnstamm was relatively small and relatively transient (Figure 4). These findings argue against a positive force feedback model.

### Opposite Pattern of Movement Velocity 400–500 ms Post-Perturbation in Kohnstamm Movements Compared to Voluntary Movements

During Voluntary movements, velocity initially increased in the Assistive perturbation condition and decreased in the Resistive perturbation condition. These changes then reversed direction after around 400 ms, showing decrease in the Assistive perturbation condition and increase in the Resistive perturbation condition (Figure 3E). Interestingly, this late reversal did not occur during Kohnstamm movements (Figure 3D), suggesting that it may reflect a voluntary response. Fitting linear trend lines to individual participant averages during this time window (400–500 ms post-perturbation) showed that the difference was statistically significant (Figure 3G). The mean value of these slopes did not differ in magnitude across movement types (*F*_(1,14)_ = 0.033, *p* = 0.859). For the main effect of Perturbation condition, Mauchly’s test indicated that the assumption of sphericity had been violated (*χ*^2^_(2)_ = 14.202, *p* = 0.001), therefore degrees of freedom were corrected using Greenhouse-Geisser estimates of sphericity (*ε* = 0.601). This resulted in there being no main effect of Perturbation condition (*F*_(1.201, 16.821)_ = 0.204, *p* = 0.702). However, there was a significant Movement type × Perturbation condition interaction (*F*_(2,28)_ = 21.621, *p* < 0.001). To explore this interaction, one-way ANOVAS were conducted. There was a significant difference across perturbation conditions in both Kohnstamm (*F*_(2,28)_ = 8.426, *p* = 0.00137) and Voluntary movements (*F*_(2,28)_ = 13.077, *p* < 0.001). Inspection of Figure 3 shows that velocity following perturbation of voluntary movements began to return towards the levels shown in No Perturbation control trials, after around 400 ms. In contrast, velocity following perturbation during Kohnstamm contractions showed a more sustained response, without return to the unperturbed levels over this time window. This produced the crossover interaction shown in Figures 3D,E,G, Planned comparisons confirmed that during Kohnstamm movements, Resistive perturbation condition velocity decreased relative to the No perturbation control condition (*t*_(14)_ = −2.420, *p* = 0.03), while Assistive perturbation condition velocity *increased* relative to No perturbation control (*t*_(14)_ = 2.162, *p* = 0.048). In contrast, for Voluntary movements, Resistive perturbation condition velocity *increased* relative to No perturbation control (*t*_(14)_ = 3.54, *p* = 0.003), while Assistive perturbation condition *decreased* relative to control (*t*_(14)_ = −2.499, *p* = 0.026). There was no significant difference in the mean slope of No perturbation control condition velocity across movement types (*t*_(14)_ = − 0.47, *p* = 0.963). Thus, the pattern of late (>400 ms) responses to perturbation was qualitatively different between Kohnstamm and voluntary movements, likely reflecting the recruitment of an additional voluntary response to the perturbation during voluntary but not Kohnstamm movements.

### Increased Kohnstamm EMG Not Explained by Behavioral Differences or Activity of Other Muscles

Across perturbation conditions, agonist EMG was higher during Kohnstamm movements compared to matched Voluntary movements (Figures 3A–C). This was not explained by differences in recorded torque during the same time window. During Kohnstamm movements, mean Resistive perturbation condition torque was 0.58 Nm (SD = 0.04 Nm), compared to 0.07 Nm (SD = 0.05 Nm) during No perturbation control condition and −0.44 Nm (SD = 0.04 Nm) during the Assistive perturbation condition. During Voluntary movements, mean Resistive perturbation condition torque was 0.63 Nm (SD = 0.11 Nm), compared to 0.13 Nm (SD = 0.10 Nm) during No perturbation control and −0.38 Nm (SD = 0.10 Nm) during the Assistive perturbation condition. Mauchly’s test indicated that the assumption of sphericity had been violated in the case of the main effect of Perturbation condition (*χ*^2^_(2)_ = 4.909, *p* = 0.086) and the Movement type by Perturbation condition interaction (*χ*^2^(2) = 6.967, *p* = 0.031), therefore degrees of freedom were corrected using Greenhouse-Geisser estimates of sphericity (Perturbation condition: *ε* = 0.761; Movement type × Perturbation condition: *ε* = 0.707). There was a significant main effect of Perturbation condition (*F*_(1.522, 21.301)_ = 18765.987, *p* > 0.001), but no main effect of Movement type (*F*_(1,14)_ = 3.634, *p* = 0.077) and no interaction (*F*_(1.414, 19.79)_ = 0.264, *p* = 0.694). Angular displacement showed the same pattern of results in this time window. Mean arm angle during Kohnstamm movements was 32.02° (SD = 10.43°) for the Resistive perturbation condition, 31.71° (SD = 9.16°) for the No perturbation control condition, and 33.62° (SD = 8.93°) for the Assistive perturbation condition. During Voluntary movements, it was 30.39° (SD = 10.92°) for the Resistive perturbation condition, 31.87° (SD = 9.73°) for No perturbation control, and 34.27° (SD = 10.37°) for the Assistive perturbation condition. There was a significant main effect of Perturbation condition (*F*_(2,28)_ = 6.2, *p* = 0.0059), but importantly, no main effect of Movement type (*F*_(1,14)_ = 0.96, *p* = 0.761) or interaction (*F*_(2,28)_ = 1.667, *p* = 0.215). For velocity of movement, there was a main effect of Perturbation condition (*F*_(2,28)_ = 26.924, *p* > 0.001) with Resistive perturbations reducing velocity and Assistive perturbations increasing velocity, as predicted (Figures 3D–F). However, again there was no main effect of Movement type (*F*_(1,14)_ = 0.304, *p* = 0.59) and no interaction (*F*_(2,28)_ = 2.038, *p* = 0.149).

**Figure 5:**
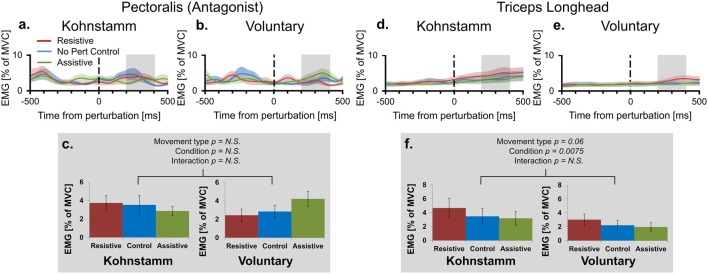
Mean smoothed antagonist and triceps long head EMG across movement types and perturbation conditions. Smoothed group (*n* = 11) antagonist EMG (% MVC) across perturbation conditions, from 500 ms prior to onset of perturbation to 500 ms post-perturbation, for Kohnstamm **(A)** and Voluntary movements **(B)**. Mean antagonist EMG (200–400 ms post-perturbation) showed there were no differences across movement types and perturbation conditions **(C)**. Smoothed group (*n* = 9) triceps long head EMG (% MVC) across perturbation conditions for Kohnstamm **(D)** and Voluntary movements **(E)**. Mean triceps long head EMG (200–400 ms post-perturbation) showed that this muscle was not more active during Voluntary than Kohnstamm movements **(F)**. There was a trend in the other direction. EMG increased in the Resistive perturbation condition and decreased in the Assistive perturbation condition, relative to the No perturbation control condition. This change in EMG was significant across the two types of movement.

Higher agonist activity in Kohnstamm movements could be due to differences in the state of antagonist muscle. However, recordings from the pectoralis showed that EMG was low and flat across all trial types (Figures 5A–C). In the time window of interest there was no main effect of Movement type on antagonist EMG (*F*_(1,10)_ = 0.114, *p* = 0.742). There was also no main effect of Perturbation condition (*F*_(2,20)_ = 0.245, *p* = 0.785) or Movement type × Perturbation condition interaction (*F*_(2,20)_ = 2.782, *p* = 0.112).

Lower agonist activity during Voluntary movements could reflect contributions of other synergist muscles to the voluntary movement. This hypothesis predicts higher activity in the triceps long head muscle during Voluntary movements than during Kohnstamm movements. In fact, we observed a trend in the opposite direction (*F*_(1,8)_ = 4.777, *p* = 0.060; Figures 5D–F). In this muscle, there was also a main effect of Perturbation condition in the same direction as for the agonist muscle (*F*_(2,16)_ = 6.739, *p* = 0.0075). Once again there was no Movement type × Perturbation condition interaction (*F*_(2,16)_ = 0.498, *p* = 0.617).

### Increased Kohnstamm EMG Not Explained by Muscle Fatigue During Kohnstamm Induction

**Figure 6:**
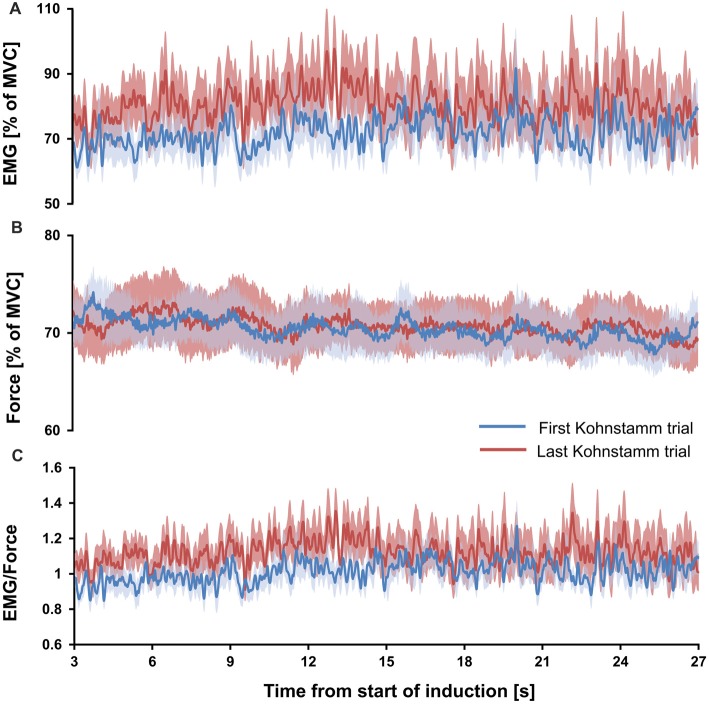
Mean EMG, force and EMG/force during first and last Kohnstamm induction period. Mean EMG (% of MVC) recorded from the posterior deltoid during the Kohnstamm induction period for the first and last Kohnstamm trial **(A)**. Mean force (% of MVC) generated by an isometric contraction of the posterior deltoid during the Kohnstamm induction period for the first and last Kohnstamm trial **(B)**. Target force was 70% MVC. Mean EMG/Force during the Kohnstamm induction period for the first and last Kohnstamm trial **(C)**. Note that there was no significant increase in the ratio of EMG to Force produced from the start of each induction to the end, nor was there a significant increase when comparing the first to the last Kohnstamm trial.

If the high EMG observed during the Kohnstamm aftercontraction was due to muscle fatigue, caused by the strong, sustained nature of the Kohnstamm induction contraction, then the ratio of EMG to force should have significantly increased, both during each induction period and also across successive inductions. However, no such increase was observed. Mean EMG to force ratio for the 1 s window at the start of first Kohnstamm induction was 0.93 (SD = 0.2) and 1.03 (SD = 0.27) at the end of the first Kohnstamm induction. During the last Kohnstamm induction it was 1.04 (SD = 0.25) at the start of the induction and 1.1 (SD = 0.46) at the end (Figure 6C). There was no significant main effect of Kohnstamm trial (*F*_(1,13)_ = 2.907, *p* = 0.112), no significant main effect of Time (*F*_(1,13)_ = 1.249, *p* = 0.284) and no significant Kohnstamm trial by Time interaction (*F*_(1,13)_ = 0.284, *p* = 0.603). Mean EMG (% of MVC) and mean force (% of MVC) for the same trials and time periods are shown for comparison (Figures 6A,B).

## Discussion

Perturbations that increased loading on the muscle during Kohnstamm aftercontraction produced an increase in EMG and a decrease in velocity. Perturbations that decreased loading produced a decrease in EMG and an increase in velocity. When EMG levels were expressed relative to MVC the size of this response did not differ from those induced during matched voluntary movements. However, after controlling for differences in the overall EMG level between unperturbed Kohnstamm and voluntary movements, the EMG response to perturbation was significantly *smaller* during Kohnstamm than during matched voluntary movements. Thus, while overall EMG levels were higher during Kohnstamm than during voluntary movement, reflex gains were lower during Kohnstamm than during voluntary movement.

The comparatively low reflex gain during Kohnstamm responses runs counter to theories of the peripheral origin of the Kohnstamm phenomenon (Hagbarth and Nordin, 1998). These theories suggest that thixotropic changes in the muscle during the induction phase lead to higher than normal levels of spindle sensitivity, which in turn drive the aftercontraction via spinal reflexes. Such models predict high afferent gains during Kohnstamm movements. Instead, we found low gains. Our results are consistent with a previous finding of *smaller* EMG stretch responses to hitting an obstacle during vertical Kohnstamm aftercontractions, compared to during voluntary movements (De Havas et al., 2015). In that study the mean stretch response (increase in EMG 60–160 ms post-obstruction) during Kohnstamm movements was 58.22% of the mean stretch response observed during voluntary movements, although this difference did not reach statistical significance. The present study with horizontal movement allowed a purer measure of response to perturbation, without the confounding effects of gravity present in earlier studies.

Our results are also difficult to reconcile with positive force feedback models of the Kohnstamm phenomenon (Parkinson and McDonagh, 2006). Positive feedback loops rapidly multiply the effects of inputs to the system, predicting large and sustained changes in EMG in response to perturbations, instead of the small and relatively transient responses we observed. Indeed, when we took the entire available time window (0–500 ms post-perturbation) we observed no significant positive or negative linear trend in Kohnstamm aftercontraction EMG in response to increased or decreased muscle loading. The EMG changes to perturbation during the Kohnstamm phenomenon were brief, despite the perturbation being present throughout this 500 ms time window.

In agreement with neuroimaging and brain stimulation studies (Mathis et al., 1996; Duclos et al., 2007; Parkinson et al., 2009; Ghosh et al., 2014), our results suggest common control mechanisms between Kohnstamm and voluntary movements. One model of voluntary motor control (Marsden et al., 1975, 1976, 1977) suggests that a central motor signal sets the equilibrium point (EP) of the muscle. This results in a follow-up servo contraction of the muscle due to negative feedback from muscle spindles, causing a movement towards the EP (Feldman, 1966; Latash, 2008b). The EP might itself move gradually over time, defining a virtual trajectory (Bizzi et al., 1984). When the muscle is stretched, as by resistive perturbation for instance, increased spindle firing causes a further contraction of the muscle, resulting in an increase in EMG, driving the arm towards the current EP. Conversely, when the existing stretch on the muscle decreases there is decreased efferent output until the spindle signal indicates that the position of the arm is returning to the current EP. It remains, however, controversial whether EP models fully explain voluntary movement (Gomi and Kawato, 1996, 1997) and the exact representation of the high level *“control signal”* (e.g., position, velocity, or force) has not been established (Kakei et al., 1999). Our results indicate that servo-control exists during the Kohnstamm aftercontraction, but with lower gain than during voluntary control. This afferent-mediated control of EMG may occur at a lower level than the Kohnstamm generator and may not alter the output of the Kohnstamm generator itself (De Havas et al., 2015; De Havas, 2016; De Havas et al., 2017). However, it be should noted that multiple neural mechanisms could be jointly involved in generating Kohnstamm aftercontractions (see for example Selionov et al., 2009, 2013; Solopova et al., 2014, 2016).

Our findings highlight an important difference between Kohnstamm and Voluntary movements, namely that the gain of the efferent and afferent arms of the control loop may be different. During the Kohnstamm phenomenon, the strong, sustained muscle contraction (Kohnstamm induction) causes a central adaptation, consisting of two components. The first component is the reduction in the gain of the afferent arm of the sensorimotor control loop, as evidenced by a reduced EMG perturbation response. The second component is higher efferent output, relative to voluntary movements. In particular, EMG levels during unperturbed Kohnstamm movements were higher than during velocity-matched voluntary movements. The increased efferent signal could arise in the putative Kohnstamm generator itself or could reflect an increased gain on the efferent arm of a sensorimotor control loop at a lower (e.g., spinal) level.

The higher EMG levels observed in Kohnstamm compared to voluntary movements may seem strange given the reported almost-linear relationship between force and EMG at a given muscle length (Calvert and Chapman, 1977; Lawrence and De Luca, 1983). Several possible explanations exist. Co-contraction of other muscles in the Kohnstamm condition might lead to higher agonist EMG. However, we found no evidence that the high EMG was caused by differences in the recruitment of other muscles (though we did not record from muscles of the back). Second, increased EMG in the Kohnstamm condition without change in torque or movement kinematics might be a result of fatigue. We cannot test this hypothesis directly, because we did not have any independent marker of fatigue. However, we found no evidence of progressive muscle fatigue within a single Kohnstamm induction, or across successive inductions, as measured by the ratio of EMG to force during the 30 s inducing contraction at 70% MVC. Thus, cumulative effects of fatigue appeared absent from our Kohnstamm condition. Absence of fatigue during such a contraction may seem surprising (Taylor and Gandevia, 2007), but it could reflect the extensive rest periods provided between trials. Central fatigue (Todd et al., 2003) is also unlikely to explain the results. Central fatigue is normally conceived as an increase in the perceived effort required to generate a given voluntary EMG. In our Kohnstamm condition, however, the EMG during the lift phase itself is perceived as effortless, even though it involves a greater EMG than a matched voluntary movement.

A third possible explanation of high aftercontraction EMG is a difference between conditions in motor unit recruitment. Even modest voluntary contractions (Suzuki et al., 1990) and muscle loads (Calancie and Bawa, 1985) lower the threshold for motor unit firing. Since motor units are recruited in size order, with smaller units recruited first (Henneman, 1985), the preceding voluntary isometric contraction in the Kohnstamm condition, would result in more motor units being recruited, and in a greater proportion of larger motor units being recruited, relative to the voluntary condition. This could explain the increased EMG in the former compared to the latter. This increased EMG might nevertheless leave movement kinematics and muscle torque unaltered, because the prolonged, strong contractions in the Kohnstamm induction phase could affect molecular processes in the muscle fiber itself (Debold et al., 2016). Most importantly, any decrease in the motor unit recruitment threshold in the Kohnstamm condition would be expected to increase both the background EMG during the Kohnstamm lift, *and also* the stretch-induced reflex EMG. This is because the recruitment order of motor units to stretch loads (Calancie and Bawa, 1985) and also to tonic vibration (Romaiguère et al., 1993) was shown to be identical to recruitment order during voluntary contractions. In fact, we found a relative *decrease* in stretch reflex EMG in the Kohnstamm, compared to the voluntary condition, suggesting an additional mechanism over and above changes in motor unit recruitment.

High levels of EMG have been reported in previous Kohnstamm studies. For example, Kohnstamm movements of lateral deltoid were previously reported to produce the same level of EMG as larger voluntary movements of the same muscle (Mathis et al., 1996). It has also been reported that there are differences in motor unit firing rates for Kohnstamm and matched voluntary movements (Kozhina et al., 1996).

High efferent output with low afferent gain is compatible with the goal of maintaining stable body posture. During normal standing or locomotion, postures are maintained for extended durations. Afferent input is relatively stable and predictable, since forces such as gravity are effectively unchanging. Under such conditions, high afferent gains may be inefficient or even detrimental to stability. Meanwhile, if the descending tonic drive was updated via voluntary control every time a new posture was adopted, the resulting attentional load would be high (Wright, 2011). As such, the Kohnstamm phenomenon may represent an extension of low-level postural mechanisms, which normally form an automatic backdrop to voluntary movements (Gurfinkel et al., 1989). Though postural control has often characterized in terms of reflexive responses to the environment, stability can also be achieved by providing a high level of tonic drive, while down-regulating afferent gains (Davidoff, 1992). When required contractions are slow and sustained, this tonic control may be more appropriate than purely reflexive, environmentally-triggered control, at least for axial and proximal muscle groups that require maintaining high levels of tonic force involuntarily, over extended time periods (Massion et al., 2004). Conversely, such control would be inappropriate for distal muscles involved in skilled dextrous actions, where responses to perturbation must rapidly restore normal muscle activity levels (Hiramatsu et al., 2015). In support of this, it has been consistently reported that the Kohnstamm phenomenon is strongest for axial and proximal muscles and weak or absent in distal muscles (Matthaei, 1924; Gurfinkel et al., 1989).

The muscle activity involved in maintaining a posture may rely on different neural circuitry than that involved in moving to a new posture (Ivanenko and Gurfinkel, 2018). Indeed, the primary motor cortex in monkeys contains distinct populations of neurons that are active ether during posture or during movement respectively (Crammond and Kalaska, 1996; Kurtzer et al., 2005). If the Kohnstamm phenomenon does represent postural activity, our findings of higher EMG and reduced reflexes relative to voluntary movement could reflect the downstream consequences of a different pattern of M1 activity across movement types. In addition, there could be important subcortical differences. Building on extensive work showing distinct control networks for eye movements, it has recently been theorized that separate “move” and “hold” systems may be a general feature of the motor system, and that the “hold” system maintains muscle activity via the sustained output of regions of the reticular nuclei that integrate commands from the “move” system (Shadmehr, 2017). The sustained command of the Kohnstamm induction might be integrated over time to produce a temporary shift in the baseline state of this “hold” system. Future research should address whether such systems underpin the putative Kohnstamm generator.

In conclusion, we used a horizontal manipulandum to study the effects of resistive and assistive perturbations on an involuntary movement, without the confounding effects of gravity. Our results showed that Kohnstamm aftercontractions involve stronger EMG drive than physically-matched voluntary contractions, coupled with a *lower* gain of the response to peripheral perturbations. Our results cannot readily be explained by previous theories attributing the Kohnstamm to increased sensitivity of muscle spindles, or to positive feedback loops driven by a muscle force signal. Rather, the Kohnstamm phenomenon involves a combination of strong, central efferent drive, with relatively weak, servo-type sensory-triggered corrective signaling. This servo control appears to operate in a similar manner to that observed during voluntary movements. That the Kohnstamm phenomenon is also characterized by high efferent drive and low afferent gain, and is relatively slow in character, may reflect its connection to postural control. Study of the Kohnstamm phenomenon may reveal the mechanisms whereby postural stability is maintained involuntarily via adaptations in response to ongoing muscle activity. This may maintain stability of the body by high tonic activity in proximal muscles, accompanied by relatively low muscle reactivity in response to changes in the environment. Whereas previous studies of postural control emphasized the importance of phasic reflex responses to sensory signals (Feldman and Mindy Levin, 1995; Marsden et al., 1976), our analysis of the Kohnstamm suggests that central, efferent mechanisms play an important role in a second, tonic aspect of postural control.

## Author Contributions

JDH, SI, PH and HG designed the research. JDH and SI performed the research. JDH analyzed the data. JDH, PH and HG wrote the article.

## Conflict of Interest Statement

HG and SI were employed by Nippon Telegraph and Telephone Corporation. The other authors declare that the research was conducted in the absence of any commercial or financial relationships that could be construed as a potential conflict of interest.
